# Circulating exosomal miR-16-5p and let-7e-5p are associated with bladder fibrosis of diabetic cystopathy

**DOI:** 10.1038/s41598-024-51451-7

**Published:** 2024-01-08

**Authors:** Bo Xue, Gaohaer Kadeerhan, Li-bin Sun, Yong-quan Chen, Xiao-feng Hu, Zi-kuan Zhang, Dong-wen Wang

**Affiliations:** 1 Shanxi Medical University, Taiyuan, 030001 China; 2 Department of Urology, National Cancer Center/National Clinical Research Center for Cancer/Cancer Hospital & Shenzhen Hospital, Chinese Academy of Medical Sciences and Peking Union Medical College, Shenzhen, 518116 China; 3 Department of Urology, First Hospital of Shanxi Medical University, Taiyuan, 030001 China

**Keywords:** Diagnostic markers, Urology, Non-coding RNAs, Endocrine system and metabolic diseases, Experimental models of disease

## Abstract

Diabetic cystopathy (DCP) is a prevalent etiology of bladder dysfunction in individuals with longstanding diabetes, frequently leading to bladder interstitial fibrosis. Research investigating the initial pathological alterations of DCP is notably scarce. To comprehend the development of fibrosis and find effective biomarkers for its diagnosis, we prepared streptozotocin-induced long-term diabetic SD rats exhibiting a type 1 diabetes phenotype and bladder fibrosis in histology detection. After observing myofibroblast differentiation from rats’ primary bladder fibroblasts with immunofluorescence, we isolated fibroblasts derived exosomes and performed exosomal miRNA sequencing. The co-differentially expressed miRNAs (DEMis) (miR-16-5p and let-7e-5p) were screened through a joint analysis of diabetic rats and long-term patients’ plasma data (GES97123) downloaded from the GEO database. Then two co-DEMis were validated by quantitative PCR on exosomes derived from diabetic rats’ plasma. Following with a series of analysis, including target mRNAs and transcription factors (TFs) prediction, hubgenes identification, protein–protein interaction (PPI) network construction and gene enrichment analysis, a miRNA-mediated genetic regulatory network consisting of two miRNAs, nine TFs, and thirty target mRNAs were identified in relation to fibrotic processes. Thus, circulating exosomal miR-16-5p and let-7e-5p are associated with bladder fibrosis of DCP, and the crucial genes in regulatory network might hold immense significance in studying the pathogenesis and molecular mechanisms of fibrosis, which deserves further exploration.

## Introduction

Diabetes mellitus (DM) is distinguished by the presence of hyperglycemia, oxidative stress and persistent inflammation, which eventually lead to systemic complications^1,2^. With the increase of incidence rate, it has brought huge burden to patients and the society^3^. Diabetic cystopathy (DCP) or diabetic bladder dysfunction (DBD) a prevalent urologic complication arised from DM. Decreased bladder sensitivity, increased urine storage volume, detrusor underactivity and poor urinary function are salient features in the late stage of the disease^4,5^. Although these patients were severely affected the quality of their life by the urological symptoms, the early pathological changes of the bladder are often difficult to detect and diagnose in the initial stages of DCP^6,7^. Detrusor instability and interstitial fibrosis of the bladder were the most prominent changes of bladder structural remodeling in DCP. The detrusor muscle of bladder undergoes the reverse process from initial compensatory hyperplasia to late decompensation; however, the bladder interstitial fibrosis is accompanied by the step-forward progress of the whole disease^8,9^, which is worth identifying as a persistent clinical characteristic.

The pathological process of fibrosis leads to loss of homeostasis and dysfunction in many tissues and organs damaged by diabetic complications, including the bladder^10–12^. Fibrosis is commonly attributed to the excessive accumulation of extracellular matrix (ECM) components subsequently contributing to scarring and perturbation of organ function. Collagen fibrils are the main component of the ECM and the collagen in bladder tissue is mainly collagen I and collagen III, with collagen I providing stretch and collagen III providing elasticity, both of which are key factors in bladder compliance^13^. With the massive deposition of collagen in the bladder wall, there is a relative decrease in elastin, which in turn leads to bladder wall hypertrophy, decreased smooth muscle function, reduced compliance and fibrosis. Bladder fibrosis occurs mainly as a result of increased synthesis and decreased degradation of the extracellular matrix, resulting in an accumulation of extracellular matrix, particularly an increase in type I and type III collagen fibres, with a predominant increase in type III collagen fibres, resulting in an increased III/I ratio^14^. Deveaud et al. also proved that the total collagen fibril content of the bladder was increased and the expression of both type I and type III collagen fibrils was upregulated in bladder fibrosis^13^. Therefore, it is hoped to better understand the disease progression of DCP by exploring the clinicopathological features based on the signatures of bladder fibrosis as a means to find effective treatments for bladder fibrosis disease markers, improvement or even reversal of fibrosis.

Exosomes, small vesicles that carry bioactive molecules including DNA, RNA and proteins, are recognized as important agents in cellular and interorgan communication^15^. Under the stimulation of different microenvironments, all types of cells can actively and specifically encapsulate miRNAs and emit them into the surrounding extracellular environment in the structure of exosomes to respond to changes^16^. With the development of disease, the components of exosomes will change. However, the protection of vesicles can delay the degradation of internal active substances, which facilitates our exploration of disease mechanisms^17^. Extensive scientific studies have uncovered the potential application of exosomes and exosomal miRNAs in molecular diagnostics, drugs delivery system and therapeutic agents for DM and associated complications^18–21^. A comprehensive understanding of exosomal contents may not only elucidate the fundamental pathogenic mechanisms of DM complications but also offer significant biomarkers and therapeutic tools for diseases. Based on the recognition of potential genetic information, our understanding of diseases involves the participation of cellular structures and intercellular communication in biological processes. Thus, it can be seen that the analysis of circulating exosomal microRNAs can provide us the early non-invasive examination of bladder remodeling signatures, especially interstitial fibrosis, which showing us with a better understanding of biological processes in DCP disease.

## Results

### Potential mechanism and study design

**Figure 1:**
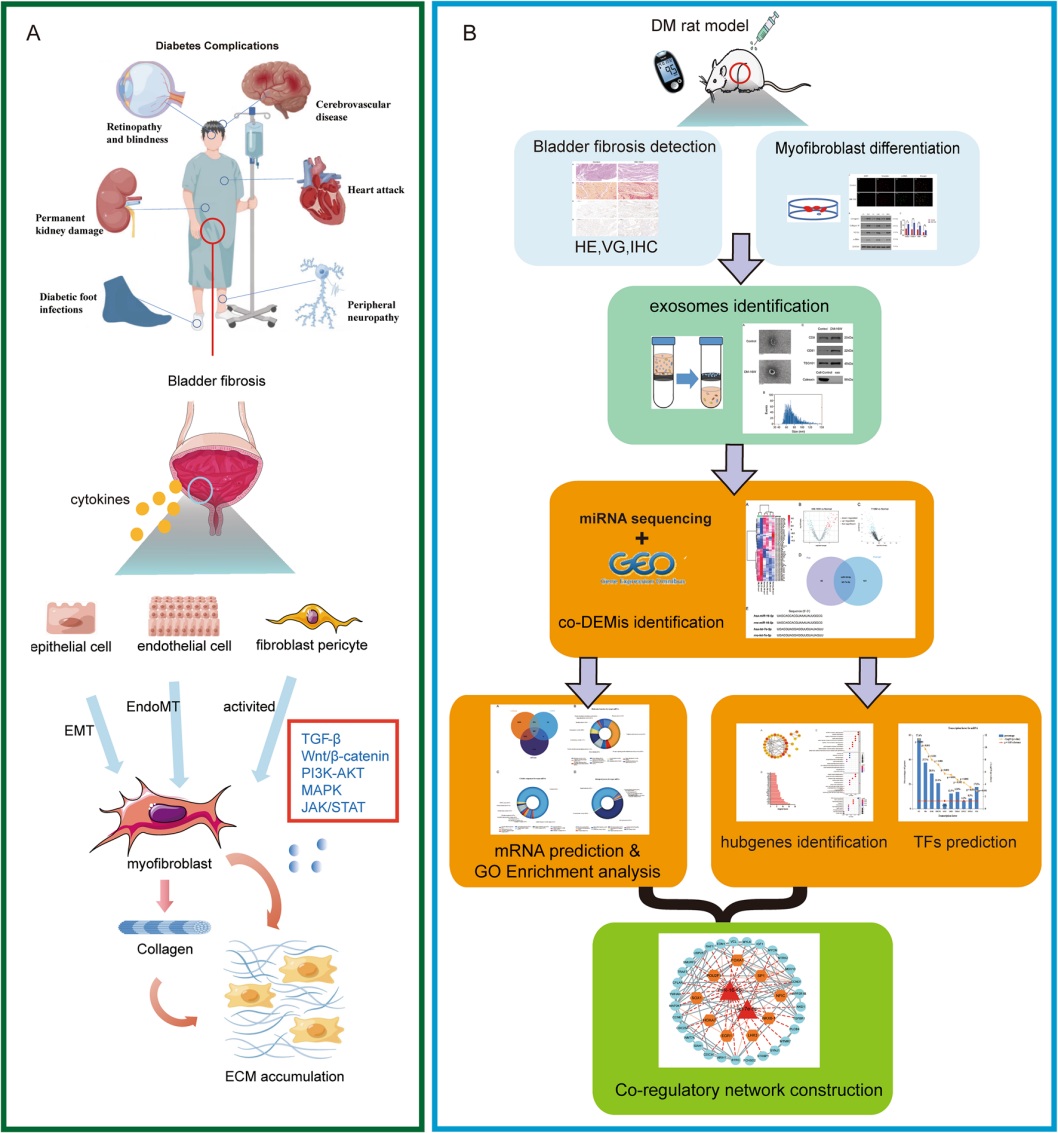
Potential mechanism of bladder fibrosis in DCP and workflow of this study. (**A**) Potential mechanism of bladder fibrosis in DCP. Inflammation and oxidative stress are triggered by high glucose stimulation in the bladder, resulting in cytokines released. As a result of severe or prolonged stimulation, fibroblasts and pericytes are activated, while bladder epithelial cells and endothelial cells experience phenotypic transformations commonly recognized as EMT and EndoMT, correspondingly, further inducing myofibroblasts formation. These processes involved various signaling pathways, including TGF-β, Wnt, MAPK, PI3K/Akt and JAK-STAT pathway. As a result of the pathological modifications, myofibroblasts are irreversibly formed along with the synthesis of a variety of collagens and accrual of ECM deposits, eventually causing the bladder interstitial fibrosis of DCP. (**B**) Workflow of this study. *EMT* epithelial-mesenchymal transition, *EndoMT* endothelial-mesenchymal transition, *ECM* extracellular matrix.

The potential mechanism of bladder fibrosis in DCP and workflow are presented in Fig. 1. Inflammation and oxidative stress are triggered by high glucose stimulation in the bladder, resulting in cytokines released. As a result of severe or prolonged stimulation, fibroblasts and pericytes are activated, while bladder epithelial cells and endothelial cells experience phenotypic transformations commonly recognized as epithelial-mesenchymal transition (EMT) and endothelial-mesenchymal transition (EndoMT), correspondingly, further inducing myofibroblasts formation. These processes involve a variety of signaling pathways, including TGF-β^22^, Wnt^23^, MAPK^24^, PI3K/Akt^25^ and JAK-STAT^26^ pathway. As a result of the pathological modifications, myofibroblasts are irreversibly formed along with the synthesis of a variety of collagens and accrual of ECM deposits, eventually causing the bladder interstitial fibrosis of DCP. In this study, we set up STZ-induced long-term diabetic rat models to comprehend the development of fibrosis in DCP through histological observations (H&E staining, VG staining and IHC). Additionally, the isolated primary bladder fibroblasts of diabetic rats were identified as the differentiation state into myofibroblasts by IF method. We carried out a joint analysis of the miRNA sequencing data for bladder fibroblasts-derived exosomes from DCP rats and plasma circulating exosomal miRNA sequencing data of patients with long-term diabetes (GES97123). The co-DEMis between diabetic rats and human (miR-16-5p and let-7e-5p) were identified. Several bioinformatics analyses were conducted to find the bioprocesses involved, including target mRNAs prediction by Targetscan, miRDB and miRWalk databases, gene enrichment analysis using ClusterProfiler package in R, hubgenes identification with degree scores calculation, protein–protein interaction (PPI) network construction and transcription factor (TF) prediction. Eventually, a co-regulatory network encompassing 2 miRNAs, 9 TFs, and 30 mRNAs was delineated; and several hubgenes may fulfill significant and multifaceted functions in the development mechanism of fibrosis, which deserves more attention for the management of DCP disease.

### Diabetes induction and fibrotic alterations in bladder of long-term diabetic rats

**Figure 2:**
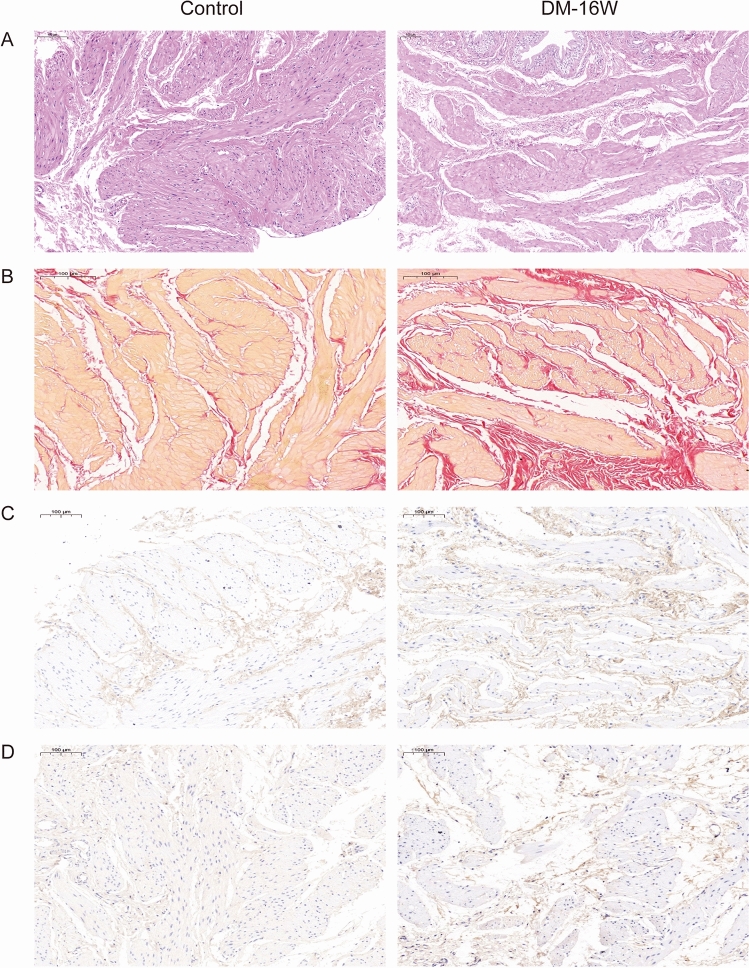
The H&E staining, VG staining and IHC staining extracted from normal control and diabetic rats. (**A**) H&E staining for bladders. Comparing with normal control group, the bladder in DM-16W group were observed looser structure with features in reduction of detrusor, a resultant widening of inter muscular spaces and the increased fibrous tissue. (**B**) VG staining for bladders. The muscle tissue is stained yellow or orange, whereas, fibrin is stained red. More area of fiber components can be observed in bladder tissue of DM-16W rats. (**C**,**D**) IHC for bladder with collagen type I and III. DM-16W rats had more positive expression of fiber components than control group, especially type I and type III collagen fibers.

Diabetes induction was validated by analyzing serum glucose and all rats in control group and DM-16W group could meet the experimental requirements. Comparing to the control rats, the diabetic rats had lower body weight, higher serum glucose levels and bladder wet weight (Supplementary Table 1). Then, we isolated the bladder tissue of rats for relevant pathological detection. Histological examination revealed that the bladders in DM-16W group were observed looser structure with features in reduction of detrusor, the resultant widening of inter muscular space and the increased fibrous tissue (Fig. 2A). As fibrosis advances, matrix in the fibrotic bladders is considerably modified compared with normal tissues from the result of VG staining (Fig. 2B). The above results were consistent with previous bladder structural changes of DCP rat models^9^. From the results of IHC, the main component of fibrous tissue was collagen type I and III (Fig. 2C,D). For both rat models’ and patients’ perspectives, extensive exposure to a diabetic environment over a prolonged period of time can trigger the progression of bladder fibrosis.

### Myofibroblast differentiation of rat primary bladder fibroblasts

**Figure 3:**
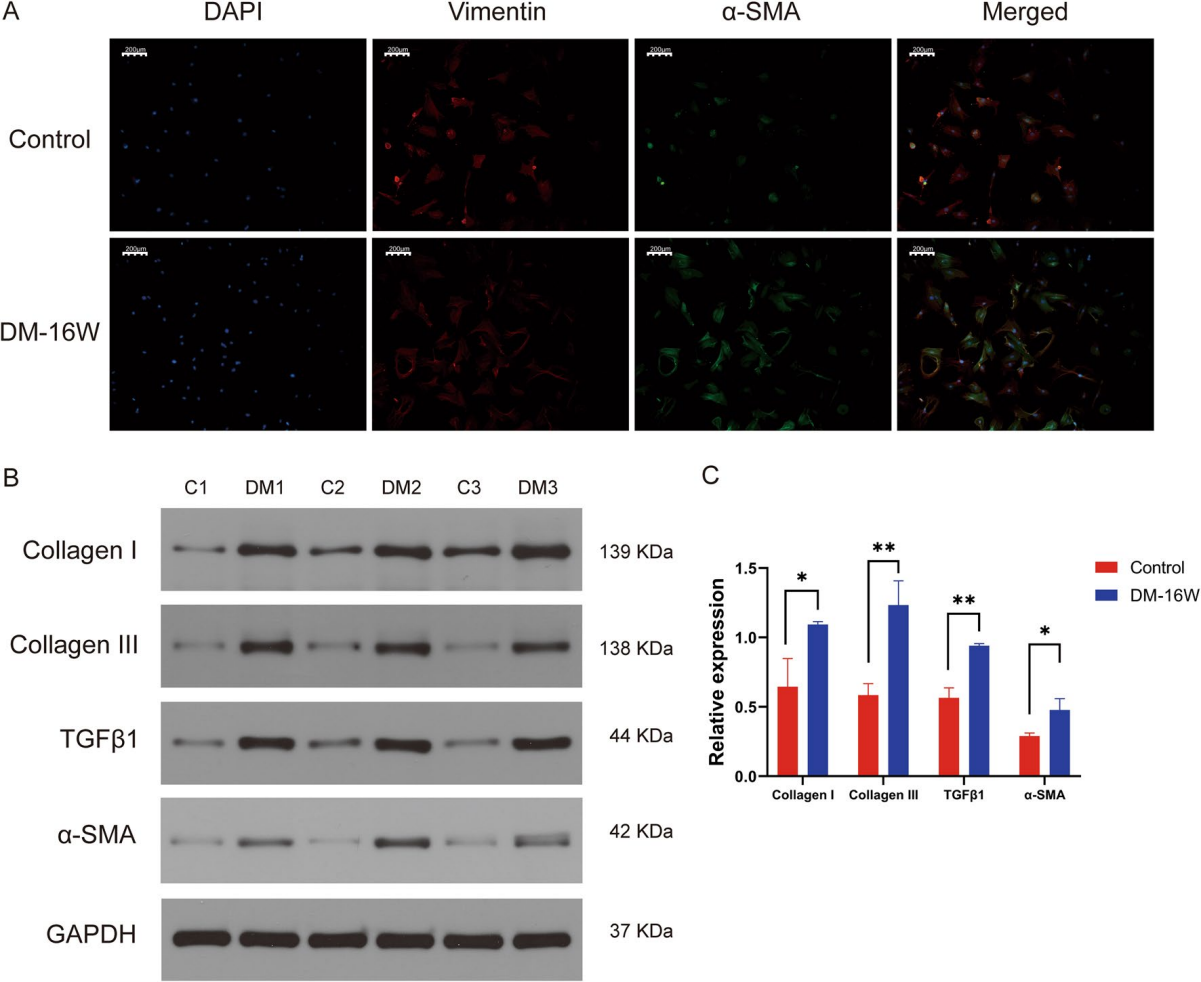
Detection of differentiation of fibroblasts into myofibroblasts. (**A**) IF for myofibroblasts in normal control group and DM-16W group. Red fluorescence (vimentin) was observed in both two groups; however, green fluorescence (α-SMA) can be seen more in DM-16W group compared to control group. (**B**) WB for Collagen I, III, TGFβ1 and α-SMA. The gel has been cropped to simplify presentation and the original gel images are shown in Supplementary Fig. 1. (**C**) Relative expression of Collagen I, III, TGFβ1 and α-SMA. The data were analyzed by unpaired Student’s *t* test. *P < 0.05, **P < 0.01.

Fibroblasts are the main component of bladder wall. Activation by environmental stimuli, fibroblasts can transform from a state of quiescence to a state of cellular proliferation during which they acquire the myofibroblast phenotype^27^. Myofibroblast is the main cell type promoting fibrosis, with a phenotype between fibroblast and smooth muscle cell. Under the induction of cytokines, including transcription growth factors (TGF-β), activated myofibroblasts expressing α-SMA protein are the main source of structural ECM proteins in fibrotic muscle tissue^28^. As shown in Fig. 3A, the vimentin protein as a marker of fibroblasts were stained fluorescent red in both control group and DM-16W group. However, there were more expression level of α-SMA protein in DM-16W group of the fibroblasts with green fluorescence stained. Besides, western-blot analysis confirmed higher expression of collagen I, III, TGFβ1 and α-SMA proteins in fibroblasts in DM-16W group compared to control group (Fig. 3B). Thus, the rat primary bladder fibroblasts differentiated into myofibroblasts phenotype when it was stimulated by high glucose.

### Identification of rat bladder fibroblast-derived exosomes

**Figure 4:**
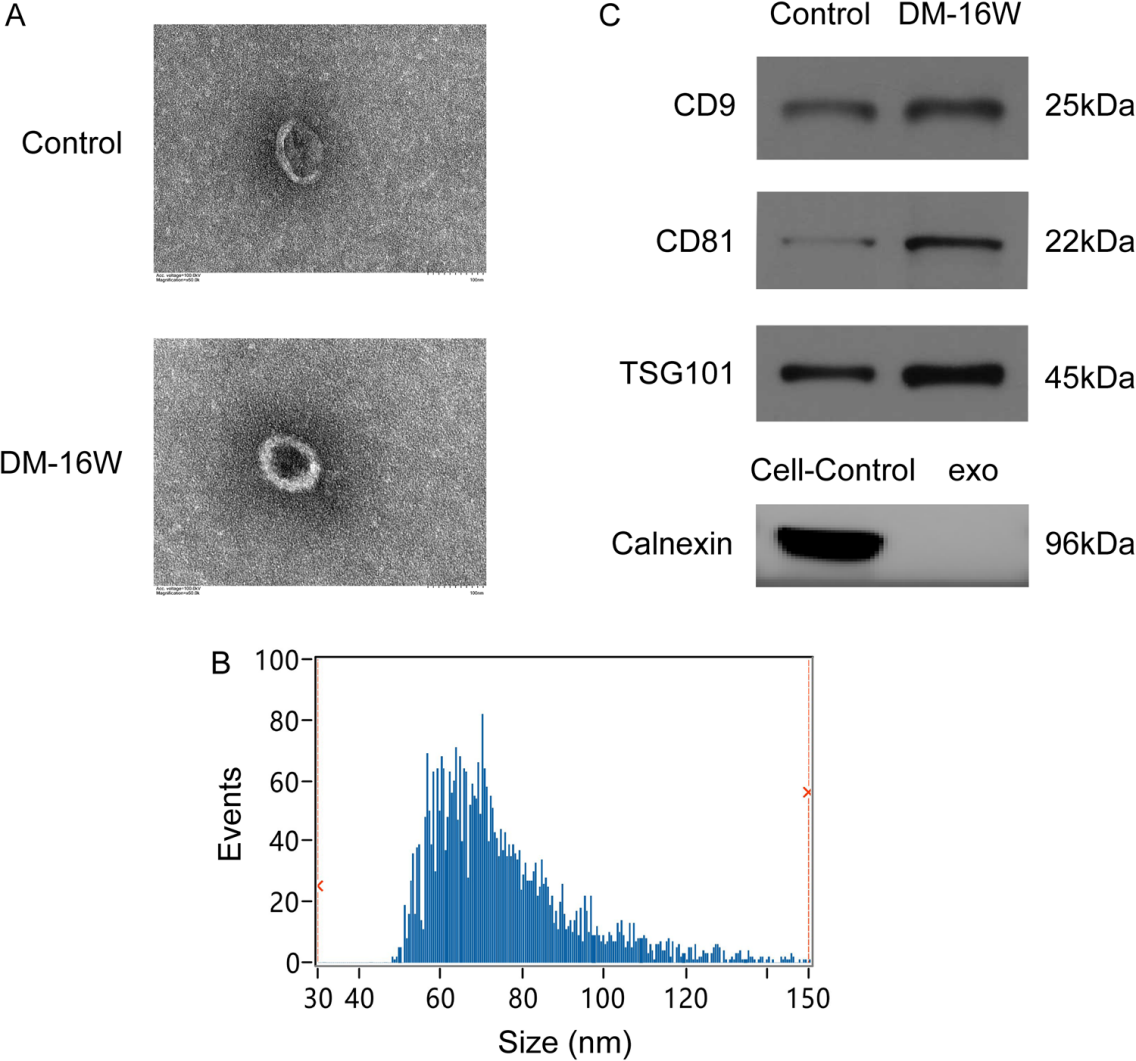
Identification of exosomes derived from rat bladder fibroblasts. (**A**) Fibroblast-derived exosomes showed the typical cup shape with transmission electron microscope (TEM) (scale bars, 100 nm). (**B**) An average size of approximately 30–150 nm by nanoparticle tracking analysis (NTA). (**C**) The representative immunoblots for exosomal marker proteins in fibroblast-derived exosomes samples from normal control and diabetic rats (DM-16W) and the negative control group contrast to fibroblasts-exosomes was the cell control. The proteins in question were detected in the exosome pellets derived from every sample. Gel images have been cropped and enhanced. The original gel images are shown in Supplementary Fig. 1B.

After differentiation to myofibroblasts, fibroblasts usually show the phenotype of contraction and synthesis. As one of the sources of exosomes secretion in microenvironment, we detected the exosomes derived from fibroblasts. The exosomes enriched from rat fibroblasts were examined by TEM and NTA, and the existence of small, round vesicles ranging in size from 30 to 150 nm were shown in Fig. 4A,B. What’s more, the validation of exosome enrichment was additionally verified through the identification of exosomal marker proteins for CD9, CD63, CD81 and TSG101 by immunoblotting (Fig. 4C). From the above results, the fibroblast-derived exosomes in both the control and DM-16W groups were successfully isolated and validated; following this, the contents inside the exosomes can be used for next research.

### Exosomal miRNA sequencing analysis for diabetic patients and rats

**Figure 5:**
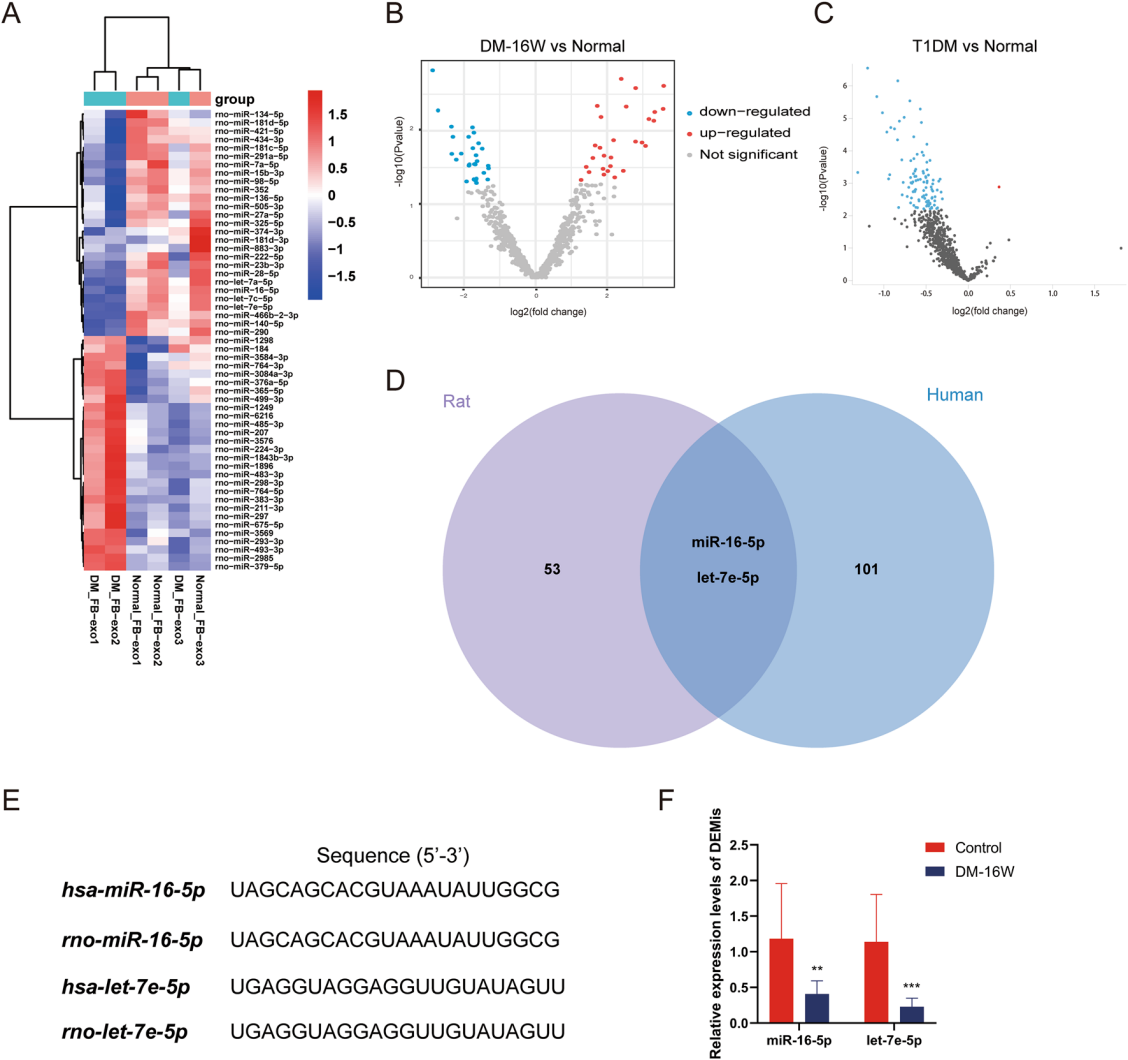
Screening and identification of bladder fibroblast derived exosomal DEMis from diabetic rats and human plasma. (**A**,**B**) Volcano plot and heat map for fibroblast-derived exosomal miRNAs in DM-16W and control rats. The DEMis were screened with edgeR package, and both |log2FC|> 1 and P < 0.05 were seen as significant. (**C**) The DEMis in plasma circulating exosomes of T1DM and normal patients were analyzed by GEO2R tool based on limma method. The Padj < 0.05 was set with significance and the results were displayed by volcano plot. (**D**,**E**) The Venn plot showed the co-DEMis between the rat and human. The sequence of rat, human miR-16-5p and let-7e-5p were completely identical. (**F**) The expression of miR-16-5p and let-7e-5p from rat’s plasma exosomes was detected in DM-16W group and control group using qPCR. **P < 0.01; ***P < 0.001(analyzed by the independent-samples *t* test).

Recently, as the active contents in exosome, miRNAs have attracted a great deal of attention on complications of diabetes. In our study, we sequenced bladder fibroblast derived exosomal miRNAs from long-term diabetic rats previously extracted; and the sequencing results of long-term T1DM patients’ plasma exosomal miRNAs were conducted a combined analysis. There were 55 differentially expressed miRNAs (DEMis) in diabetic rats and 103 DEMis in diabetic patients compared to the normal samples respectively (Fig. 5A–C). After taking the intersection of both candidate miRNAs lists, two co-DEMis (miR-16-5p and let-7e-5p) were screened out (Fig. 5D). What’s more, the sequences of these two miRNAs from rat were identical with the corresponding target sequence from human species (Fig. 5E). Then the expression of co-DEMis from plasma exosomes of rats was performed qPCR for validation. These two co-DEMis were downregulated in DM-16W group comparing with control group. What are the functions of these two DEMis and whether they play a significant role in disease progression? This warranted further investigation.

### Target mRNA predictions and GO enrichment analysis

**Figure 6:**
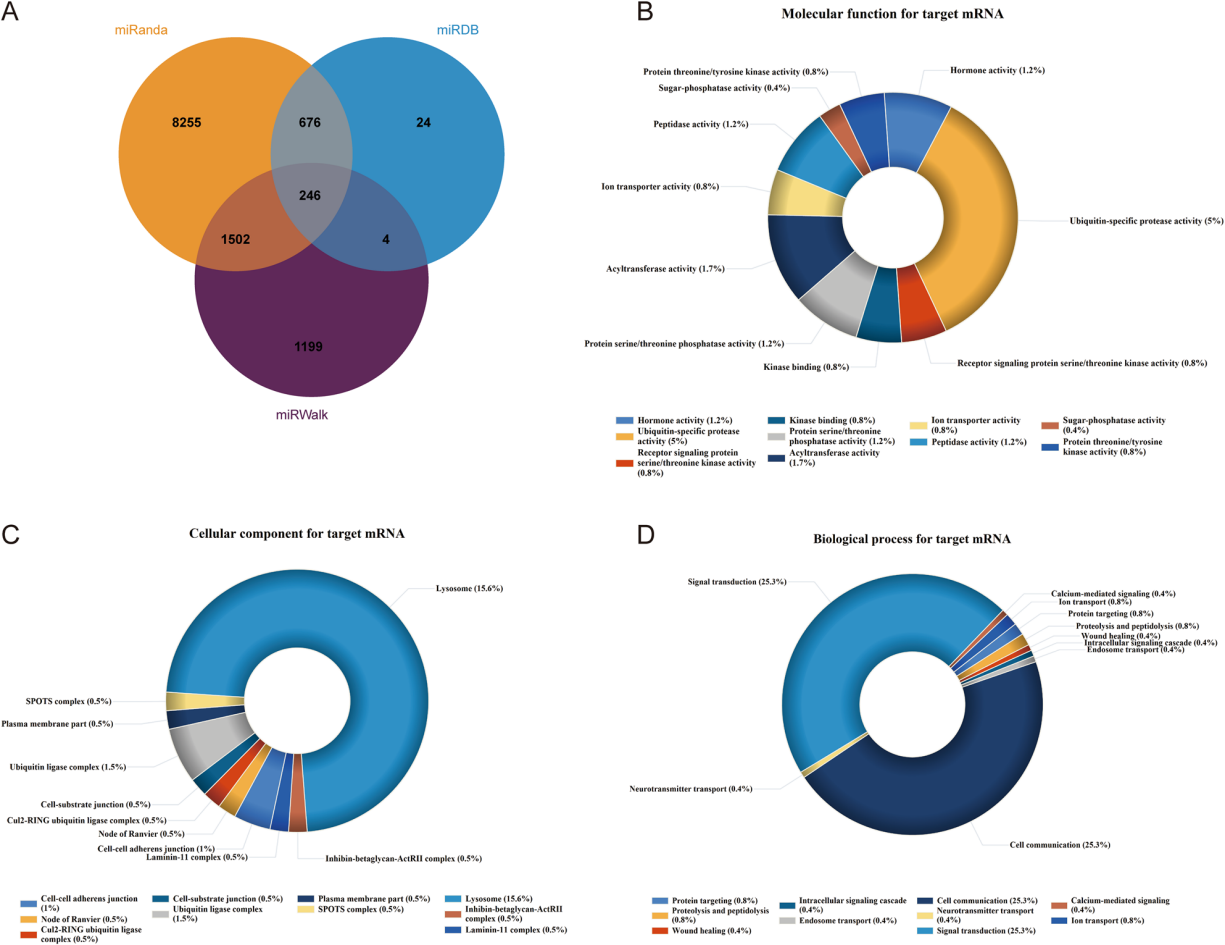
Prediction for target mRNA and GO Enrichment analysis of exo-DEMis. (**A**) Target mRNAs prediction for exo-DEMis. We took the intersection of target mRNA predicted by miRanda, miRDB and miRWalk database for miR-16-5P and let-7e-5P. The intersection was drawn in Venn plot. (**B**–**D**) GO Enrichment analysis for 246 target mRNAs.

Typically, miRNAs are chiefly involved in the biological regulation process via their associations with the corresponding target mRNAs. We screened out 246 potential target mRNAs of DEMis with several prediction algorithms, such as miRanda, miRDB and miRWalk. Regarding the molecular function (MF) terms identified through GO enrichment analysis of 246 target mRNAs, a majority of the genes were found to be associated with ubiquitin-specific protease activity (5%), acyltransferase activity (1.7%), protein threonine/tyrosine kinase activity (1.2%), hormone activity (1.2%), peptidase activity (1.2%) (Fig. 6B). The analysis of GO enrichment has also brought to light that the top three cellular component (CC) terms exhibit the most enriched targets of DEMis include lysosome (15.6%), ubiquitin ligase complex (1.5%) and cell–cell adherens junction (1%) (Fig. 6C). The five most enriched targets of DEMis in terms of biological progress (BP) were confirmed to be the cell communication (25.3%), signal transduction (25.3%), proteolysis and peptidolysis (0.8%), protein targeting (0.8%) and ion transport (0.8%) (Fig. 6D). Accordingly, 246 target mRNAs of miR-16-5p and let-7e-5p were screened by using bioinformatics, and these identified target genes were particularly related to a broad range of biological processes.

### Identification and enrichment analysis of hubgenes

**Figure 7:**
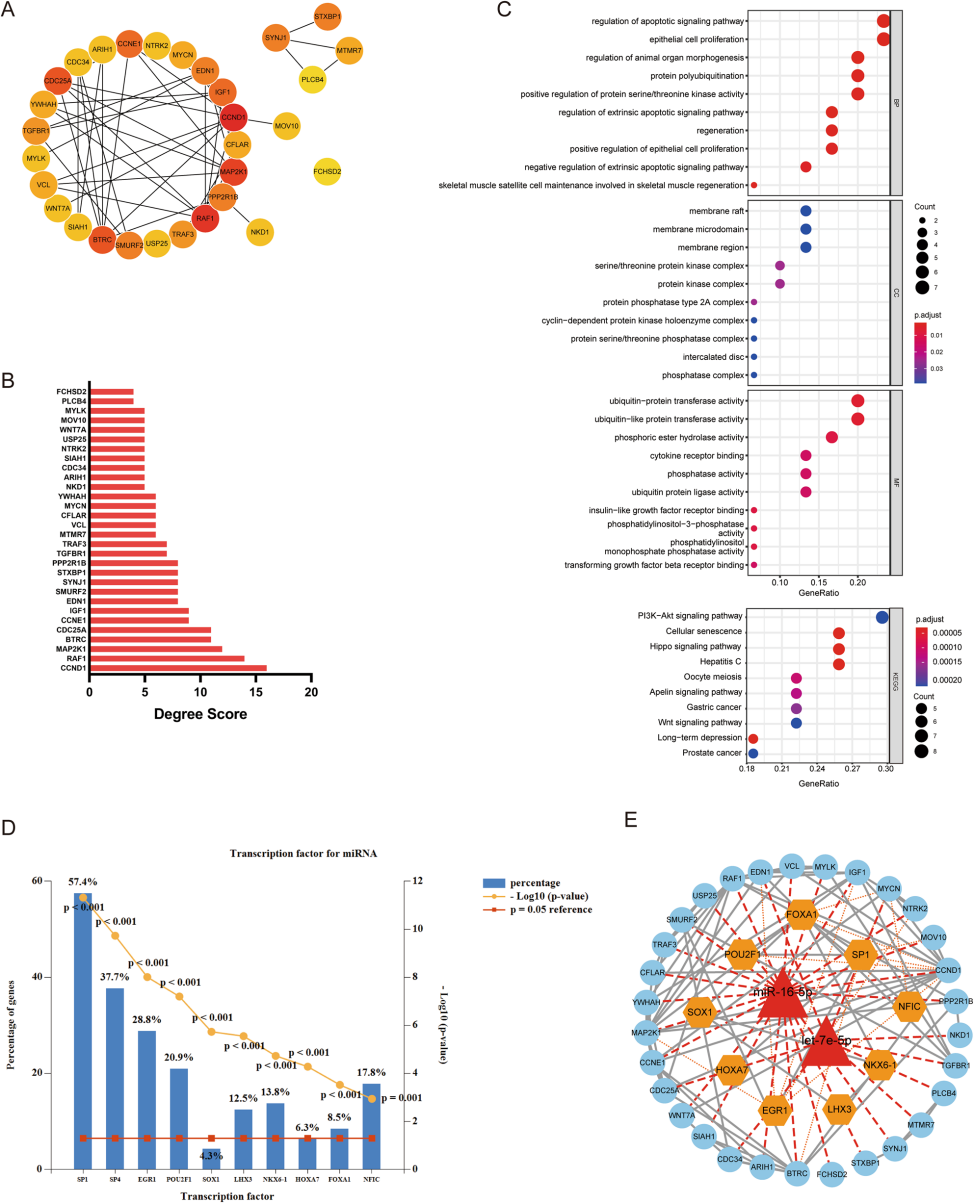
Hubgenes enrichment analysis, transcription factor prediction and TFs-miRNA network construction. (**A**,**B**) The nodes of target mRNAs were linked by Cytoscape software and the nodes in red color represent hub genes and edges represent the interactions between nodes. The top 30 hubgenes were ranked by scores computed with the degree algorithm. (**C**) GO and KEGG enrichment analysis of 30 hubgenes. (**D**) Transcription factor prediction for miR-16-5P and let-7e-5P based on Funrich software. TOP 10 TFs with P < 0.05 were shown in plot. (**E**) The construction of regulatory network among miRNAs, mRNAs and TFs. The TF-miRNA-mRNA regulatory network was constructed using Cytoscape software. Only the nodes with edges were shown in the network. The hierarchical relationships between the miRNAs and mRNAs were shown with red lines; and orange line indicated the regulatory relationship of TFs-mRNAs.

To further explore the underlying molecular mechanism, we used Cytoscape software to construct a PPI network derived from the STRING database. The interactions between 30 hubgenes calculated with degree method were illustrated in Fig. 7A, and the 30 highest-ranking genes based on the number of degree scores were displayed with the bar plot (Fig. 7B). As shown in Fig. 7C and Supplementary Table 2, most of the genes were involved in regulation of apoptotic signaling pathway, protein polyubiquitination, positive regulation of protein serine/threonine kinase activity, regeneration and positive regulation of epithelial cell proliferation in the BP term. As to CC term, membrane part, serine/threonine protein kinase complex, phosphatase complex and cyclin-dependent protein kinase holoenzyme complex were most activited. From the result of MF term, most genes were involved in ubiquitin-protein activity, cytokine receptor binding, phosphatidylinositol-3-phosphatase activity and phosphatase activity. Furthermore, it was found that 10 KEGG pathways were enriched, including PI3K-Akt signaling, Apelin signaling, Hippo signaling, Wnt signaling, Cellular senescence, Long-term depression, Oocyte meiosis. The results described above further enlightened the major biological pathways and relevant regulatory processes of hubgenes in more detail.

### Transcription factor prediction and the construction of TF-miRNA-mRNA co-regulatory network

In addition, transcription factors (TFs) can activate or suppress miRNA expression, whereas miRNAs can silence mRNAs encoding TFs. To understand the crosstalk network between the TFs and miRNA-mRNA regulations, two DEMis and 246 target genes were mapped into 198 TFs. Following an investigation into the enrichment of targets of TFs, we filtered the leading 10 TFs which had strong closeness to miRNAs, including SP1, SP4, EGR1, POURF1, SOX1, LHX3, NKX6-1, HOXA7, FOXA1, and NFIC (Fig. 7D), suggesting these TFs being involved in regulatory interactions with DEMis. Next, 10 TFs and DEMis-target 30 hubgenes were submitted to STRING database to explore a close connection with each other. As a result, we get a miRNA-mediated genetic regulatory network with 2 miRNAs, 9 TFs and 30 target mRNAs regulation relationship among them (Fig. 7E). The target genes which were co-regulated by miRNA and TFs were listed in Supplementary Table 3, including CCND1, MAP2K1, BTRC, IGF1, EDN1, MYCN, NTRK2, MOV10 genes. TFs, miRNAs and target mRNAs in this regulatory network were the contents we need to focus on.

## Discussion

Recently, a large number of studies have shown that exosomal miRNAs play a significant role in mediating fibrosing diseases progression^29–31^. Given the advantages of integrity of containing contents, noninvasive and convenient for detection, many researchers have focused on exploring the application of exosomal miRNA as a tool for diagnosis and treatment of fibrosis. We set up STZ-induced long-term DM models of SD rat to explore the potential mechanism of bladder fibrosis and the experimental flow was shown in Fig. 1. It can be observed that the content of collagen fibers in bladder were significantly increased and disorganized. Additionally, the isolated primary bladder fibroblasts were identified as the differentiation state into myofibroblasts from DCP rats. We carried out a joint analysis of the miRNA sequencing data for bladder fibroblasts-derived exosomes from DCP rats and plasma circulating exo-miRNA sequencing data of patients with long-term diabetes. The co-DEMis between diabetic rats and human (miR-16-5p and let-7e-5p) were identified. A series of bioinformatics analyses inspired us that these two DEMis, corresponding target mRNAs and TFs might contribute to the process of fibrosis. Eventually, we described TF-miRNA-mRNA co-regulatory network composed of 2 miRNAs, 9 TFs and 30 mRNAs; and several hubgenes may play important, multidimensional roles in the development mechanism of fibrosis, which deserves more attention for the management of DCP disease.

With the incidence of diabetes increasing yearly, diabetic cystopathy, as a common urologic complication, gains more attentions in diagnosis and treatment of disease. When the disease progresses to advanced stage, the lower urinary tract symptoms of DCP patients are obviously observed, which affecting their quality of life. Although the research on the pathogenesis of DCP involves oxidative stress, bladder structural remodeling, peripheral neuropathy, urinary tract epithelial changes and urine stimulation, etc.^4,5,9^, there is currently a lack of reliable and unified diagnostic standard for disease. Moreover, due to the poor awareness of the need for medication, the patients with DCP usually received no definitive diagnosis and little treatment. As a kind of structural bladder cystopathy, detrusor instability and interstitial fibrosis of the bladder were the most remarkable alterations in DCP. The bladders from rats subjected to cystometry testing were further dissected and their weights were then measured. Gross histological examination suggested that disturbance of tissue architecture consist of less detrusor smooth muscle and increased fibrous tissue were observed in DM-16W rats compared to normal rats (Supplementary Table 1, Fig. 2). Next, after VG staining for bladder tissues, it can be seen that the area of fibrous tissue represented by yellow was significantly increased. The common fibrosis indicators including several collagen types, cohesin, TGFβ, hyaluronidase^32,33^, etc. were selected to detected by IHC. The above results reflected that we have successfully constructed the DCP rat model, and also observed the bladder fibrosis.

To further observe fibrotic phenotype, we isolated primary bladder fibroblasts and used IF microscopy to identified the reliable marker for fibroblasts (vimentin). Interestingly, in addition to the observation of vimentin in DM-16W rats and normal rats, we also identified α-SMA representing muscularized component in the DM group. The transformation of fibroblasts into myofibroblasts expressing α-SMA protein under the stimulation of cytokines, such as TGF-β, cells with mesenchymal properties that are crucial to the secretion and remodeling of the ECM, is a common symptom of fibrosis^28,34^. Then, we conducted WB analysis to confirm the aforementioned conjecture. Elevated levels of Collagen I, III TGF-β1 and α-SMA were observed in DM-16W group compared to the control group (Fig. 3). The above findings supported the myofibroblast trans-differentiation of rat bladder fibroblasts under high glucose condition. This particular transformation process usually affects the stiffness and composition of the tissue in an autocrine and/or paracrine manner^35^. Lv et al. built up the model rats with interstitial cystitis (IC) by instilling exosomes released by transduced ADMSCs into the bladders of postmenopausal rats^30^. Their team discovered that the promotion of the EMT process and resultant bladder wall fibrosis leading to IC in postmenopausal women is facilitated by the increase in Mfn2 which results from the suppression of miR-214. However, the upregulation of miR-363 in STZ-induced T2DM rats can reduce detrusor fibrosis by inhibiting TGF-β1/Smad signaling pathway^36^. It can be seen that the bidirectional regulation of the fibrotic process through exosomes communication results from the properties of the miRNA it carries.

Importantly, we identified exosomes secreted by rat bladder fibroblasts with TEM, NTA and representative immunoblots, and analyzed the contents of exosomes by the high-throughput sequencing. The electron micrograph and NTA showed the presence of 30-150 nm sized small, round vesicles along with the exosomal marker proteins immunoblotted in both control and DM-16W groups (Fig. 4). The combined analysis for rat bladder fibroblasts derived exosomal miRNAs and long-term DM patients’ plasma exosomal miRNAs revealed that two co-expressed miRNAs (miR-16-5p and let-7e-5p) with the corresponding target sequence in both patients and rats were screened (Fig. 5). It has been reported a reduction in miR-16-5p expression and an increase in NOTCH2 expression among systemic sclerosis patients. Additionally, in vitro experiments confirmed the direct binding of miR-16-5p to NOTCH2, leading to the suppression of myofibroblast activation^37^. Exosomes derived from keratinocytes which were rich in miR-16-5p were found to inhibit the enhancement of fibroblast proliferation, migration, and Col1a1 expression induced by TGF-β1^38^. As reported, the biological functions and regulation of miR-16-5p are mainly enriched in insulin-like growth factor-I (IGF-I), which is closely related to insulin resistance^39,40^. What’s more, miR-16-5p was also detected as a molecular marker in exosomes of urine from diabetic nephropathy^21^. Usually, the *Let-7* family of miRNAs have a profound impact on the control of glucose homeostasis and insulin sensitivity^41^. Limited research has been conducted on the biological function of miRNA let-7e-5p. Suli Huang et.al proposed the potential use of let-7e-5p as a biomarker for ischemic stroke and showed that it is involved in related pathological mechanisms^42^. A study has shown that the expression changes of 11 miRNAs (including let-7e-5p) may be related to low-grade chronic inflammation and oxidative stress in patients with hyperglycemia^43^. Liu et al. found that let-7e-5p may have a similar regulatory effect in the rat model of nonalcoholic liver fibrosis^44^. Summarizing, miR-16-5p is known to be the most important microRNA involved in insulin resistance pathway in many tissues during diabetes confirming the diabetic states. There are few reports exploring the connections between let-7e-5p and diabetes, more to ischemia and inflammation. It can be seen that these two DEMis may play a regulatory role in biological progress as potential markers for fibrosis disease through their interactions with the associated target mRNAs.

Besides, miR-16-5p has been identified as a tumor suppressor in various tissues, with the exception of ovarian cancer tissues^45–49^. Furthermore, several studies have observed up-regulation of long non-coding RNAs (lncRNAs) that target miR-16-5p or its specific targets^50,51^. These observations highlight the intricate network through which miR-16-5p exerts its tumor suppressor effects. Silva et al. proposed a new signature of four circulating miRNAs, including let-7e-5p, as a potential predictive tool for detecting colorectal cancer^52^. The roles of these two miRNA biomarkers in DCP patients deserve further exploration, which also requires more experiments. 246 target genes of two DEMis were identified through intersection of multiple databases for target prediction (Fig. 6). Then, we performed GO enrichment analysis for these mRNAs, and obtained the gross domains of bio-function for target genes. Most target genes were involved in ubiquitination process (post-translational regulation of proteins and misfolded protein recognition), regulatory mechanism of protein kinase activity, protein synthesis and breakdown, cell–cell adherens junction, cell communication and signal transduction. These biological processes are involved in the pathogenesis of many diseases including fibrosis. Dysregulation of ubiquitination^53^ and EMT^54^, characterized by the dissolution of cell–cell adhesion have been observed in the development of fibrotic diseases.

Based on the above-mentioned background, the present study focused more attention on the core genes identification and underlying molecular mechanism exploration in this context. The cytoHubba plugin of Cytoscape software was employed to obtain the degree scores of 246 genes, and the top 30 genes ordered by scores were listed (Fig. 7B). The result of GO and KEGG pathway enrichment analysis for 30 hubgenes revealed that the regeneration and regulation of epithelial cell proliferation, ubiquitination process, phosphatidylinositol-3-phosphatase activity were the mainly enriched GO terms. Presently, the EMT of tubular epithelial cells (TECs) has been noted in renal fibrosis, and the heightened prevalence of transcription factors related to EMT influenced the advancement of the disease^55–57^. The regeneration and regulation of epithelial cells proliferation promotes EMT and interstitial fibrosis^58,59^. Besides, ubiquitin–proteasome system (UPS) serves as an exceedingly efficient and precise mechanism for modifying protein degradation^60^. There appears to be a correlation between the dysfunction of UPS components and the fibrosis progression of DN^61^. Such as UPS9X, a conserved deubiquitinase, has the capability to deubiquitinate and stabilize Smurf1, thus negatively regulating the pro-fibrotic TGF-β pathway^62,63^, thereby impacting the EMT process of renal TECs in DN^64^. Furthermore, these hubgenes were also enriched in PI3K-Akt pathway, Hippo pathway, Wnt pathway and Apelin signaling pathway from the KEGG pathway analysis results (Fig. 7C). These pathways and fibrosis diseases are closely intertwined, such as diabetic cardiomyopathy^65–67^, DN^68,69^ and other diabetic complications. The key genes that play a regulatory role in these GO terms and pathways deserve our attention.

Considering the interaction between TFs and miRNA expression, we conducted two DEMis and target mRNAs into TFs database to understand the crosstalk network between the TFs and miRNA-mRNA regulations. Base on the construction of a miRNA-mediated genetic regulatory network with 2 miRNAs, 9 TFs and 30 target mRNAs regulation relationship, the target genes which were co-regulated by miRNA and TFs were identified, including CCND1, MAP2K1, BTRC, IGF1, EDN1, MYCN, NTRK2, MOV10 (Fig. 7E, Supplementary Table 3). As for the hub genes, many studies have linked them to fibrosis diseases. For instance, cyclin D1 (CCND1), a key regulator for cell cycle and proliferation is identified the vital role of NFκB-JMJD3-CCND1 signaling in cystitis induced bladder reconstruction, as it regulates hBSMC proliferation and ECM deposition^70^. The insulin family comprises insulin, insulin-like growth factor 1, 2 (IGF-1, 2), their receptors and associated binding proteins. Elevated levels of both IGF-1 and IGF-1 receptor were observed in the glomeruli of rats with diabetes and function as profibrotic agents greatly inducing the proliferation of fibroblasts^71^. IGF-1 has the potential to enhance glomerular volume, thereby elastically stretching the mesangial cells, resulting in an augmented production of TGF-β^72^. Furthermore, the IGF receptor was also observed to facilitate the formation of α-SMA fibers in injured keratocytes, and to stimulate their differentiation into myofibroblasts^73^. The endothelin system evokes a spectrum of physiological effects in various tissues throughout the entirety of the organism. The derivation of the three ligands (EDN-1, -2, and -3) is from a substantial precursor molecule referred to as preproendothelin. EDN-1 can work in conjunction with a plethora of molecules, which are deemed pivotal in the context of fibrogenesis, such as TGF-β, platelet-derived growth factor and basic fibroblast growth factor to facilitate the process of cellular replication or transformation^74^. For instance, endothelial cells can be seen to undergo a profibrogenic cytokine TGF-β-induced increase in EDN-1 gene expression and protein release, resulting from the regulation of gene promoter via direct transcriptional mechanisms^75^. Knocking down MYCN downstream-regulated gene-2 expression in renal TECs leads to increased fibrosis as a result of its influence on EMT marker expression and ECM components through regulating the TGF-β1 pathway^76^. TGF-β1 has the capacity to impede the proliferation of B cells and the differentiation of lymphocytes. It can also govern the transcription of interleukin-6 gene in fibroblasts, expedite the expression and breakdown of extracellular matrix components, such as collagen and fibronectin, and serves as a significant indicator of renal fibrosis^77^.

Taken together, exosome-derived miRNAs can mediate the proliferation and differentiation of fibroblasts and induce EMT progress via multiple signaling pathways, thus playing a regulatory role in fibrosis diseases. The future of exosomal miRNAs research is exciting, promising a new class of diagnostic markers and therapeutic targets. Exosomes can transmit the miRNA carried to the receptor cells through paracrine or autocrine ways, and play a role in promoting fibrosis or anti-fibrosis in the target cells, suggesting that exosomes can also be used as a biological carrier of genetic material and drugs delivery to present a novel research direction for the management of fibrosis diseases.

This study had some limitations. Firstly, further elucidation is necessary regarding the fundamental mechanisms of fibrosis process of DCP have to be further explained, requesting the construction of the triplex network for miRNAs, transcription factors and mRNAs. Secondly, the potential origins of myofibroblast differentiation can be fibroblasts, detrusor cells, endothelial cells and pericytes, among others. Under the stimulation of high glucose, these cells might transform into myofibroblasts and obtain fibroblast-like phenotype, a process required further discussion. While the integrated network we constructed describes the miRNAs, TFs and target genes, the nature of the regulatory interactions remains unknown. Thirdly, the indicative role of exosomal miRNAs from urine for bladder fibrosis status might be more advocated. Urine, as a bodily fluid in direct contact with bladder tissue, may be more likely to carry signals of microenvironmental changes. This study explores based on existing plasma samples of long-term DM models of SD rat and patients. It could be interesting in the future to compare plasma and urine content of the exosomal miRNAs. As a result of insufficient empirical verification, additional investigation is required to discern the validity of these conclusions.

In this study, we have successfully identified the myofibroblast differentiation phenotype of bladder fibroblasts and have constructed a co-regulatory network involving TFs, miRNAs, and mRNAs. Specifically, we found that two miRNAs, namely exosomal miR-16-5p and let-7e-5p, are down-regulated in samples of DCP. Furthermore, we have observed that the downstream genes, particularly the 30 hubgenes, regulated by these two differentially expressed miRNAs are closely associated with the fibrosis process. This includes their involvement in fibrin, cytoskeleton, lysosome, intercellular communication, and other cellular structures that play crucial roles in various biological processes. These structural and functional changes contribute to aberrant cell proliferation and differentiation, UPS activity, ion transport disorder, EMT and other pathological processes in disease progress. It indicates that miR-16-5p and let-7e-5p have potential as bladder fibrosis markers of DCP disease, and the crucial genes in the regulatory network are of great significance in studying the pathogenesis of fibrosis, although the predicted results still need to be rigorously validated by experiments.

## Materials and methods

### Construction of long-term diabetic rat models

Eight-week-old female Sprague–Dawley (SD) rats weighting 230 ± 20 g (Animal Experiment Center of Zhuhai Chinese Academy of Advanced Technology, Zhuhai, China) were randomly allocated into two distinct groups, namely, diabetic and age-matched control rats (DM-16W and Control groups). The induction of diabetes was carried out through an intraperitoneal injection of streptozotocin (STZ) at a concentration of 60 mg/kg of body weight, freshly prepared using citrate buffer at a pH of 4.5. Conversely, the control rats received only citrate buffer. The diagnosis of diabetes was confirmed through the evaluation of blood glucose levels, which exceeded 300 mg/dl following the administration of STZ injection for 72 h. In order to avoid the development of severe hyperglycemia, ketonuria, and weight loss, administration of insulin via subcutaneous injection (up to a maximum of 2 units) was performed as needed, with the aim of promoting gradual weight gain and preventing excessive hyperglycemia. The rats were acclimated under standard laboratory conditions (ventilated room, 25 ± 1 °C, 50–60%% humidity, 12 h light/dark cycle) for 16 weeks.

At the end of the experiment, all rats were anesthetized and then sacrificed by decapitation to obtain blood and bladder tissues for a series of experiments and all animal experiments in the present study followed the Guidelines for Care and Use of Laboratory Animals of the experimental animal center in the local (TOP-IACUC-2021-0127). The study was reported in accordance with ARRIVE guidelines^78^.

### Bladder histology detection

According to established protocols, Hematoxylin–eosin (H&E) staining and Van Gieson’s (VG) staining were executed for histology observation. Briefly, paraffin-embedded bladder tissues of rats were dewaxed, rehydrated, H&E or VG stained, dehydrated. After neutral gum sealing pieces, sections were observed under a microscope. Collagen fibers were stained red by acid fuchsin, and muscle fibers were stained yellow by Picric acid. For immunohistochemistry (IHC) staining, after undergoing overnight deparaffinization and rehydration, the paraffin sections were subjected to boiling antigen retrieval buffer (ARB) at 120 °C for 5 min. Follow by adding 3% hydrogen peroxide, the sections were incubated with primary antibodies against TGFβ1, Collagen I and Collagen III (ab215715, ab270993 and ab7778, respectively, Abcam) and secondary antibodies, and the intended color was observed with diaminobenzidine (DAB) under the microscope.

### Isolation of primary fibroblasts and identification of myofibroblast differentiation

Primary fibroblasts were obtained from rats’ bladders by combined collagenase/pancreatin digestion and cultured in Dulbecco’s modified Eagle’s medium, high glucose (DMEM-HG, Gibco) containing 10% FBS. The cells were passaged to the third generation for subsequent experiments. *Immunofluorescence (IF) analysis*: Fibroblasts were inoculated into 24-well plates at a density of 1 × 10^5 cells per well with slides putting in wells already. Slides were fixed with 10% formalin, treated with 0.2% TritonX-100 for 5 min and blocked with 3% BSA, respectively. Then, the slides were incubated with a 1:200 dilution of primary antibody against α-SMA and Vimentin (GTX100034, GTX100619, GeneTex) followed by appropriate anti-rabbitsecondary antibody (GTX213110-05). Following the washing step in PBST after incubating with DAPI (GTX30920) for 10 min, anti-fluorescence quenching sealing tablets were incorporated and subsequently secured. The images were acquired by a fluorescence microscope. *Western blotting (WB) analysis*: Total protein of fibrosis related proteins was isolated from fibroblasts, and normalization of the total protein concentration was achieved via BCA assay. Subsequently, the protein samples underwent loading before being subjected to 10% SDS-PAGE and eventually transferred onto a PVDF membrane. The primary antibodies against TGFβ1, Collagen I and Collagen III were utilized to incubate the PVDF membrane at 4 °C overnight along with HRP-conjugated secondary antibodies (AS1093, Aspen) performed for an hour. An enhanced chemiluminescence system was used to visualize the specific protein bands.

### Exosome isolation and characterization identification

Fibroblasts were cultured in serum-free DMEM beforehand for 48 h. After collecting the conditioned medium, exosomes were extracted according to the instructions of an exosome extraction kit (S601, Sfj Technology Co. Ltd). Then, the transmission electron microscope (TEM) was used to observe the morphology of exosomes, and a nanoparticle tracking analyzer (NanoFCM, N30E, Xiamen) was used to identify the particle size, concentration and distribution of exosomes.

The WB analysis of total exosome protein isolated from the culture supernatant of fibroblasts was performed as described in protocol before. The usual exosomal marker proteins such as CD9, CD81, TSG101 and Calnexin (ab275018, Abcam) were detected in this step.

### Data resource and the identification of differentially expressed exosomal miRNAs

In order to identify circulating exosomal miRNAs of bladder fibrosis, we performed high-throughput sequencing for exosomal miRNAs from DCP rat’s fibroblasts. A series of annotation process included the following steps: (a) Pruning and filtering reads data with Cutadapt software. (b) Alignment of read sequences (reads) with FANSe3 algorithm^79,80^ (Rat mature miRNA, miRBase release 22.1). (c) Measures of gene expression abundance in RPM (RPM = readCount*1,000,000/libsize, libsize: sum of mapped readCount). Principal component analysis (PCA) and Pearson correlation were used to detect differences and correlations. (d) The differentially expressed miRNAs (DEMis) from DM-16W rats compared to normal control group were screened with edgeR^81^ method. Both |log2FC|> 1 and P < 0.05 were seen as significant.

For horizontal comparison of gene expression changes between cells and blood, we listed “Diabetes” and “exosomal miRNA” as keywords to search for genomewide expression studies in Gene Expression Omnibus (GEO) database to match clinical data samples. Only the datasets containing normal samples and T1DM samples with circulating exo-miRNAs expression information were the first choice for inclusion. Finally, the dataset of GSE97123 was obtained from the GEO database. The incidence of DCP among patients enduring diabetes for a duration surpassing 10 years stands at roughly 25% (up to 50% of patients with diabetes over 15 years)^82^. Considering 24 patients in the cohort had diabetes more than 25 years, all samples were included in further analysis. The DEMis in plasma circulating exosomes of T1DM and normal patients were analyzed by GEO2R tool based on limma method. Padj < 0.05 was set with significance and the results were displayed by volcano plot using ggplot2 package.

### Validation of exosomal DEMis

The blood samples of rats were collected in EDTA-K2 tubes and subjected to centrifugation at 2000*g* for 10 min at 4 °C. The supernatant was transferred into a new tube, and then centrifuged at 10,000 g for 10 min at 4 °C. The exosomes were isolated using the exosome extraction kit (S801, Sfj Technology Co. Ltd, China) and combined with ultracentrifugation^83^. In brief, (a) Add an equal volume of Isolation Regent A solution into the supernatant for coupling proteins and nucleic acids. (b) Mix and stand at 4 °C for 5 min, and centrifuge 10,000*g* for 2 min. (c) Transfer the supernatant into a new centrifuge tube containing an equal volume of Isolation Regent B solution combining of Reagent A to form a pore structure (150–220 nm). (d) Mix and stand at 4 °C for 2 h, and ultracentrifuge 13,500*g* (Optima XPN-100, Beckman Coulter, USA) 4 °C for 1 h to collect the precipitate, which is the exosome. (e) After carefully removing supernatants, the plasma exosome pellet was resuspended in an appropriate volume of PBS. The isolated exosomes can be used for subsequent experiment after characteristic identification. The process of extracting exosomal RNA from plasma samples in rats involved the use of TRIzol reagent for RNA extraction and quantitative PCR. The miRNAs first-strand cDNA and q-PCR kits were acquired from TIANGEN (Beijing, China). The forward primers used were: rno-miR-16-5p: 5′-TAGCAGCACGTAAA-3, rno-let-7e-5p: 5′-TGAGGTAGGAGGTTGT-3′ and U6 snRNA: 5′-CTCGCTTCGGCAGCACA-3′. The qPCR detection kit supplied the reverse primers. The calculation of miRNA expression relative to the expression of U6 was performed with 2^–ΔΔCt^ method.

### mRNA target prediction and GO enrichment analysis for the target of transcription

The miRanda, miRDB, and miRWalk databases were utilized to predict the potential target mRNAs of DEMis, and only the target mRNA occurred in three prediction algorithms were included in subsequent analyses. The DEMis were uploaded onto the software platform, FunRich (version 3.1.3), for Gene Ontology (GO) enrichment analysis, which is a widely utilized instrument for conducting functional enrichment analysis of enriched target genes associated with transcription factor pathways, based on its internal database^84^. According to the integrated information of miRNA and potential target mRNAs, GO enrichment analysis was then employed to establish an interaction network analysis among miRNAs, mRNAs and transcription factors.

### PPI network construction

246 target mRNAs were uploaded to STRING^85^online database (http://string-db.org) to search co-expressed genes. 165 nodes with interaction score larger than 0.4 were selected to construct protein–protein interaction (PPI) network by Cytoscape software (version 3.7.2; https://cytoscape.org/)^86^. The connectivity degree of each node was calculated by cytoHubba plugin, and top 30 nodes were shown in the plot with counts from low to high.

### GO and KEGG enrichment analyses on hubgenes

Hubgenes were subjected to Go and Kyoto Encyclopedia of Genes and Genomes (KEGG) Pathway^87^ analyses using clusterProfiler^88^, enrichplot and ggplot2 packages in the R software. Only P value < 0.05 was set statistically significant.

### Construction of TF-miRNA-mRNA co-regulatory network

All of the DEMi-mRNA pairs and TF-mRNA pairs chosen above were placed in the TF-miRNA-mRNA network, and the visualization of the network was conducted with Cytoscape software. Simultaneously, the degrees of nodes, as well as their closeness and betweenness measures within the regulatory network, were computed.

### Statistical analysis

The data were shown as the mean ± SEM. GraphPad Prism 9 software was used to perform unpaired Student’s *t* test to analyze differences between two groups. The false discovery rate (FDR) was controlled for multiple comparisons, and the data with a value of P or adjusted P < 0.05 were considered significant difference.

### Approval for animal experiments

The protocol was approved by the Institutional Animal Care and Use Committee of Shenzhen TopBiotech Co., Ltd. (approval number TOP-IACUC-2021-0127).

### Supplementary Information

Supplementary Information.

## Data Availability

Publicly available datasets were analyzed in this study. Complete microarray data can be accessed at Gene Expression Omnibus (Accession Number GSE97123).
