# Supplementary material for: Circulating exosomal miR-16-5p and let-7e-5p are associated with bladder fibrosis of diabetic cystopathy
Source: Sci Rep. 2024 Jan 8;14:837. doi: 10.1038/s41598-024-51451-7 (PMC10774280; doi:10.1038/s41598-024-51451-7)

Supplementary Materials

|  | | Control (n = 5) | DM-16W (n = 5) | |
| --- | --- | --- | --- | --- |
| Serum glucose (mmol/L) | Before diabetes induction | 6.1±0.5 | | 6.2±0.4 |
|  | At the timepoint of euthanization | 5.2±0.4 | | 24.6±2.5^*^ |
| Body weight (g) | Before diabetes induction | 234.3±19.3 | | 224.0±12.0 |
|  | At the timepoint of euthanization | 296.7±40.1 | | 185.3±15.1^*^ |
| Bladder wet weight (mg) |  | 184.7±42.7 | | 350.0±56.2^*^ |
| Bladder wet weight/ Body weight (mg/g) |  | 0.6±0.1 | | 1.8±0.2^*^ |

**Supplementary Table 1.** Changes in serum glucose, body weight and bladder weight levels in the experimental groups.

DM, diabetes mellitus. *, P < 0.05.

**Supplementary Table 2.** GO analysis and pathway enrichment analysis of 30 genes.

| **Category** | **Term ID** | **Description** | **P-Value** | **geneID** |  |  |
| --- | --- | --- | --- | --- | --- | --- |
| Biological process | GO:2001233 | regulation of apoptotic signaling pathway | 3.02E-06 | RAF1, IGF1, PPP2R1B, TGFBR1, CFLAR, YWHAH, SIAH1 |  |  |
|  | GO:0050673 | epithelial cell proliferation | 5.54E-06 | CCND1, MAP2K1, BTRC, IGF1, TGFBR1, CFLAR, WNT7A |  |  |
|  | GO:2000027 | regulation of animal organ morphogenesis | 2.54E-06 | EDN1, SMURF2, TGFBR1, CFLAR, NKD1, WNT7A |  |  |
|  | GO:0000209 | protein polyubiquitination | 1.35079E-05 | BTRC, SMURF2, TRAF3, ARIH1, CDC34, SIAH1 |  |  |
|  | GO:0071902 | positive regulation of protein serine/threonine kinase activity | 1.46731E-05 | CCND1, RAF1, MAP2K1, IGF1, EDN1, TGFBR1 |  |  |
|  | GO:2001236 | regulation of extrinsic apoptotic signaling pathway | 5.25709E-06 | RAF1, IGF1, PPP2R1B, TGFBR1, CFLAR |  |  |
|  | GO:0031099 | regeneration | 1.49858E-05 | CCND1, MAP2K1, IGF1, CFLAR, WNT7A |  |  |
|  | GO:0050679 | positive regulation of epithelial cell proliferation | 1.89382E-05 | CCND1, IGF1, TGFBR1, CFLAR, WNT7A |  |  |
|  | GO:2001237 | negative regulation of extrinsic apoptotic signaling pathway | 2.39228E-05 | RAF1, IGF1, TGFBR1, CFLAR |  |  |
|  | GO:0014834 | skeletal muscle satellite cell maintenance involved in skeletal muscle regeneration | 1.46377E-05 | IGF1, WNT7A |  |  |
| Cellular component | GO:0045121 | membrane raft | 0.001525058 | SMURF2, PPP2R1B, TGFBR1, CFLAR |  |  |
|  | GO:0098857 | membrane microdomain | 0.00154213 | SMURF2, PPP2R1B, TGFBR1, CFLAR |  |  |
|  | GO:0098589 | membrane region | 0.001776412 | SMURF2, PPP2R1B, TGFBR1, CFLAR |  |  |
|  | GO:1902554 | serine/threonine protein kinase complex | 0.000338296 | CCND1, CCNE1, TGFBR1 |  |  |
|  | GO:1902911 | protein kinase complex | 0.000534128 | CCND1, CCNE1, TGFBR1 |  |  |
|  | GO:0000159 | protein phosphatase type 2A complex | 0.000382647 | PPP2R1B, NKD1 |  |  |
|  | GO:0000307 | cyclin−dependent protein kinase holoenzyme complex | 0.001974968 | CCND1, CCNE1 |  |  |
|  | GO:0008287 | protein serine/threonine phosphatase complex | 0.002767432 | PPP2R1B, NKD1 |  |  |
|  | GO:0014704 | intercalated disc | 0.002767432 | VCL, YWHAH |  |  |
|  | GO:1903293 | phosphatase complex | 0.002767432 | PPP2R1B, NKD1 |  |  |
| Molecular function | GO:0004842 | ubiquitin-protein transferase activity | 6.28E-05 | BTRC, SMURF2, TRAF3, ARIH1, CDC34, SIAH1 |  |  |
|  | GO:0019787 | ubiquitin-like protein transferase activity | 8.24E-05 | BTRC, SMURF2, TRAF3, ARIH1, CDC34, SIAH1 |  |  |
|  | GO:0042578 | phosphoric ester hydrolase activity | 0.000301259 | CDC25A, SYNJ1, PPP2R1B, MTMR7, PLCB4 |  |  |
|  | GO:0005126 | cytokine receptor binding | 0.000941874 | SMURF2, TGFBR1, TRAF3, CFLAR |  |  |
|  | GO:0016791 | phosphatase activity | 0.001021628 | CDC25A, SYNJ1, PPP2R1B, MTMR7 |  |  |
|  | GO:0061630 | ubiquitin protein ligase activity | 0.001241688 | BTRC, SMURF2, ARIH1, SIAH1 |  |  |
|  | GO:0005159 | insulin−like growth factor receptor binding | 0.000305614 | IGF1, YWHAH |  |  |
|  | GO:0004438 | Phosphatidylinositol-3-phosphatase activity | 0.00034601 | SYNJ1, MTMR7 |  |  |
|  | GO:0052744 | phosphatidylinositol monophosphate phosphatase activity | 0.000388866 | SYNJ1, MTMR7 |  |  |
|  | GO:0005160 | transforming growth factor beta receptor binding | 0.00069722 | SMURF2, TGFBR1 |  |  |
| KEGG pathway | hsa04218 | Cellular senescence | 5.32E-07 | CCND1, RAF1, MAP2K1, BTRC, CDC25A, CCNE1, TGFBR1 | |  |
|  | hsa04390 | Hippo signaling pathway | 5.55E-07 | CCND1, BTRC, PPP2R1B, TGFBR1, YWHAH, NKD1, WNT7A | |  |
|  | hsa05160 | Hepatitis C | 5.55E-07 | CCND1, RAF1, MAP2K1, PPP2R1B, TRAF3, CFLAR, YWHAH | |  |
|  | hsa04730 | Long-term depression | 1.31E-06 | RAF1, MAP2K1, IGF1, PPP2R1B, PLCB4 | |  |
|  | hsa04114 | Oocyte meiosis | 3.46E-06 | MAP2K1, BTRC, CCNE1, IGF1, PPP2R1B, YWHAH | |  |
|  | hsa04371 | Apelin signaling pathway | 4.89E-06 | CCND1, RAF1, MAP2K1, TGFBR1, MYLK, PLCB4 | |  |
|  | hsa05226 | Gastric cancer | 7.30E-06 | CCND1, RAF1, MAP2K1, CCNE1, TGFBR1, WNT7A | |  |
|  | hsa04151 | PI3K-Akt signaling pathway | 1.26E-05 | CCND1, RAF1, MAP2K1, CCNE1, IGF1, PPP2R1B, YWHAH, NTRK2 | |  |
|  | hsa04310 | Wnt signaling pathway | 1.41E-05 | CCND1, BTRC, NKD1, SIAH1, WNT7A, PLCB4 | |  |
|  | hsa05215 | Prostate cancer | 1.42E-05 | CCND1, RAF1, MAP2K1, CCNE1, IGF1 | |  |

**Supplementary Table 3.** Target genes co-regulated by miRNAs and TFs.

| Target gene | miRNA | TF |
| --- | --- | --- |
| CCND1 | miR-16-5p | SP1, EGR1, POU2F1, FOXA1 |
| MAP2K1 | miR-16-5p | EGR1 |
| BTRC | miR-16-5p | SP1 |
| IGF1 | miR-16-5p | EGR1 |
| NTRK2 | miR-16-5p | EGR1 |
| MOV10 | miR-16-5p | SP1 |
| EDN1 | let-7e-5p | EGR1 |
| MYCN | let-7e-5p | SP1, FOXA1, NFIC |

**Supplementary Figure 1.** Original gel images


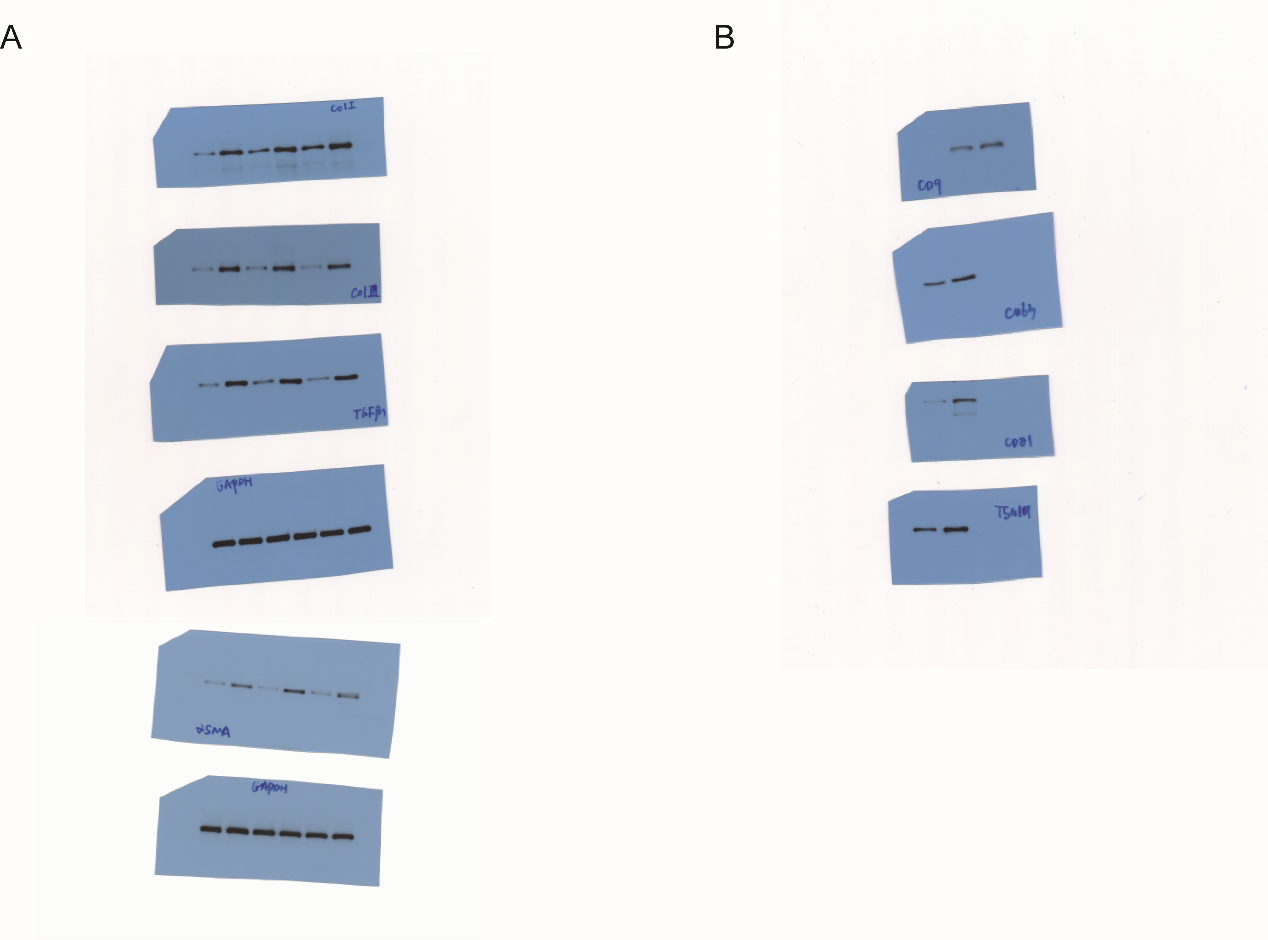

Supplement: Supplementary file 1 — Supplementary Information. [file 41598_2024_51451_MOESM1_ESM.docx]
